# MDMA‐assisted psychotherapy for the treatment of PTSD: A systematic review and meta‐analysis of randomized controlled trials (RCTs)

**DOI:** 10.1002/npr2.12485

**Published:** 2024-10-09

**Authors:** Ghada Shahrour, Kainat Sohail, Safa Elrais, Muhammad Hamza Khan, Javeria Javeid, Khubaib Samdani, Hajra Mansoor, Syed Izhar Hussain, Dhruvikumari Sharma, Muhammad Ehsan, Abdulqadir J. Nashwan

**Affiliations:** ^1^ Department of Community and Mental Health Nursing, Faculty of Nursing Jordan University of Science and Technology Irbid Jordan; ^2^ College of Nursing RAK Medical and Health Sciences University Ras Al‐Khaimah; ^3^ Department of Psychiatry Jinnah Sindh Medical University Karachi Pakistan; ^4^ Department of Psychiatry University of Tripoli Tripoli Libya; ^5^ Department of Psychiatry Karachi Medical and Dental College Karachi Pakistan; ^6^ Department of Psychiatry Allama Iqbal Medical College Lahore Pakistan; ^7^ Department of Psychiatry Rawalpindi Medical College Rawalpindi Pakistan; ^8^ Department of Psychiatry CMH Lahore Medical College Lahore Pakistan; ^9^ Department of Psychiatry Khyber Medical University Peshawar Pakistan; ^10^ Avalon University School of Medicine Willemstad Curaçao; ^11^ Department of Psychiatry King Edward Medical University Lahore Pakistan; ^12^ Hamad Medical Corporation Doha Qatar

**Keywords:** MDMA‐AT, meta‐analysis, post‐traumatic stress disorder, RCTs

## Abstract

**Background:**

Post‐traumatic stress disorder (PTSD) is a mental health disorder resulting from exposure to traumatic events, manifesting in various debilitating symptoms. Despite available treatments, many individuals experience inadequate response or significant side effects. Previous reviews suggest promising outcomes with MDMA‐assisted psychotherapy (MDMA‐AT), but limitations prompt the need for a comprehensive evaluation.

**Methods:**

We searched various online databases and registries such as MEDLINE (via PubMed), Embase, the Cochrane Central Register of Controlled Trials (CENTRAL), and ClinicalTrials.gov to retrieve RCTs that fit our inclusion criteria. We performed meta‐analyses using Review Manager by applying a random‐effects model. Dichotomous and continuous outcomes were pooled as risk ratios (RR) and standard mean difference (SMD), respectively.

**Results:**

Nine studies with a total of 297 participants with PTSD were included in our meta‐analysis. The control group consisted of inactive doses of MDMA (25–40 mg) or placebo. Our meta‐analysis showed that MDMA‐AT led to a significant reduction in the Clinician‐Administered PTSD Scale for DSM‐5 (CAPS‐5) severity scores as compared to the control group (SMD −1.10, 95% CI: −1.62 to −0.59). More patients in the MDMA‐AT group exhibited significant response (RR 1.59, 95% CI: 1.22, 2.08) and remission (RR 2.32, 95% CI: 1.47 to 3.66) as compared to patients in the control group. There was no significant difference regarding the incidence of ≥1 treatment‐emergent adverse events (TEAE), ≥1 severe TEAE, and suicidal ideation between the two groups.

**Conclusion:**

MDMA‐AT demonstrates significant efficacy in improving PTSD symptoms, enhancing both response and remission rates in individuals with chronic, treatment‐resistant PTSD, while maintaining a favorable safety profile.

## Abstract 2

We performed an updated systematic review and meta‐analysis to determine the efficacy and safety of MDMA‐Assisted Psychotherapy (MDMA‐AT) in PTSD. MDMA‐AT demonstrates significant efficacy in improving PTSD symptoms, enhancing both response and remission rates in individuals with chronic, treatment‐resistant PTSD, while maintaining a favorable safety profile.

## Graphical abstract

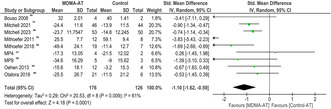

## 1 INTRODUCTION

Post‐traumatic stress disorder (PTSD) is a chronic mental condition that can occur in individuals who experience or witness a traumatic event.^1^ The typical symptoms of PTSD exemplified in intrusion, avoidance, arousal, and alterations in cognition and mood make this disorder a debilitating mental health problem with immeasurable social and economic costs.^2^ The lifetime prevalence of PTSD is estimated between 2% and 8% and this variation relates to the severity of the experienced trauma and the geographical location of the affected population.^3^, ^4^, ^5^ According to a more recent study, PTSD prevalence was estimated at 3.9% worldwide, with high‐income countries suffering the highest proportion of PTSD cases.^6^ PTSD has been associated with various physical and mental health problems including substance use, suicidality, and exacerbation of chronic illnesses such as diabetes and cardiovascular disease.^7^, ^8^, ^9^

Trauma‐focused psychotherapies including exposure and cognitive behavioral therapies are effective in the reduction of PTSD symptoms and improvement of individual functioning^10^; however, many participants fail to respond to them or continue to experience PTSD symptoms, in addition to the high dropout rate from these treatment modalities.^11^, ^12^, ^13^ The selective serotonin reuptake inhibitors sertraline and paroxetine, the Food and Drug Administration (FDA) approved drugs for the treatment of PTSD, were also found to result in a considerable rate of non‐responders. An estimated 35%–60% of patients with PTSD are not benefiting from these medications.^14^, ^15^, ^16^ In 2017, the FDA granted Breakthrough Therapy designation to a new drug for treatment‐resistant PTSD, namely amphetamine 3,4‐methylenedioxymethamphet‐amine (MDMA).^17^ Despite being a schedule I controlled substance with high abuse potential,^18^ this medication has received recent attention as a possible novel treatment for PTSD.

MDMA works by increasing the availability of the monoamine neurotransmitters, serotonin, norepinephrine, and dopamine at the synaptic cleft.^18^ The rapid increase of these neurotransmitters, especially serotonin, is thought to contribute to the pro‐social effects of the drug, including improved empathy and trust and an increased sense of social closeness and inclusion.^19^, ^20^ MDMA also facilitates the reprocessing of traumatic memories through the reduction of fear associated with revisiting these memories. MDMA accomplishes this by upregulating or restoring normal levels of brain‐derived neurotrophic factor (BDNF) in key brain regions such as the amygdala, ventromedial prefrontal cortex (vmPFC), and hippocampus. Adequate BDNF availability in these regions is crucial for memory consolidation and disrupting the association of fear‐inducing memories, thereby reducing emotional reactivity associated with traumatic experiences.^18^, ^21^ These effects are thought to contribute to the efficacy of MDMA‐assisted psychotherapy (MDMA‐AT) in PTSD patients.

Preliminary findings of interventional studies showed that MDMA‐AT can lead to a reduction in patients' PTSD symptoms and improve overall functioning.^22^, ^23^, ^24^ Our systematic review seeks to address critical gaps in the existing literature on MDMA‐AT for PTSD treatment. Previous meta‐analyses have shown promising results, indicating that MDMA‐AT reduces PTSD symptoms in severe and treatment‐resistant patients, with manageable adverse events.^25^, ^26^, ^27^, ^28^, ^29^ These analyses, though, were limited by small sample sizes and the inclusion of quasi‐experimental studies, casting doubt on the reliability of their conclusions. Even the most recent meta‐analysis by Smith and colleagues,^26^ which included six randomized controlled trials (RCTs), was limited by the small sample sizes in five of these trials. Given the publication of three recent RCTs, two of which had large sample sizes^22^, ^30^ since the last meta‐analysis, our study aimed to provide more robust evidence regarding the efficacy and safety of MDMA‐AT in PTSD treatment by incorporating these new RCTs. This will offer valuable insights for clinical practice and guide future research in this crucial area of mental health intervention.

## 2 MATERIALS AND METHODS

This systematic review was conducted following the recommendations of the Cochrane Handbook for Systematic Reviews of Interventions^31^ and reported according to the Preferred Reporting Items for Systematic Reviews and Meta‐Analyses (PRISMA) statement.^32^ The protocol was registered with the International Prospective Register of Systematic Reviews (PROSPERO: CRD42023394142).

### 2.1 Eligibility criteria

#### 2.1.1 Inclusion criteria

1. Study design: RCTs.
2. Patient population: patients diagnosed with PTSD using the Clinician‐Administered PTSD Scale for DSM‐4 and DSM‐5 (CAPS‐4and CAPS‐5).
3. Intervention: MDMA‐AT.
4. Control: Placebo/no treatment/standard treatment/inactive doses of MDMA (<40 mg).
5. Outcome: at least one outcome of interest reported.

#### 2.1.2 Exclusion criteria

1. Non‐comparative studies (e.g., case reports, case series) and observational studies.
2. Non‐randomized trials such as quasi‐experimental studies.
3. Trials that did not assess any of our pre‐defined outcomes.

### 2.2 Information sources and search strategy

We searched the following online databases and registries from 1990 to March 2024: MEDLINE (via PubMed), Embase, the Cochrane Central Register of Controlled Trials (CENTRAL), and ClinicalTrials.gov. In addition, the World Health Organization International Clinical Trials Registry Platform (ICTRP), ProQuest Dissertations and Theses Global (PQDT), and OpenGrey were used to search the gray literature for relevant studies. We also screened the reference lists of the relevant articles and previous systematic reviews to retrieve any relevant RCTs. Forward citation searching was also performed using the Web of Science to identify further eligible articles. Our detailed search strategy is given in the Table S1.

### 2.3 Selection process

Rayyan was used to remove duplicate data and to screen all the articles gathered via the online literature search. Following deduplication, two authors carried out the initial phase of screening titles and abstracts. The same two authors then performed the full‐text screening of the remaining articles. Any conflict between them was resolved by a third author. A PRISMA flow chart was constructed to illustrate the study selection process.

### 2.4 Data collection process and data items

Two authors independently extracted data from the included studies into a well‐structured Excel sheet. Relevant data items were extracted, including study characteristics (first author, year of study, trial name, location of study, study design, number of participants, duration of follow‐up), patient characteristics (age, sex, comorbid anxiety and depression, prior report of MDMA use, duration and baseline severity of PTSD, response and remission criteria), intervention details (dosage of MDMA, number of MDMA‐AT sessions and intervals between the doses), comparator details (dose of MDMA, placebo, standard treatment), and the outcomes. Our primary outcome was a change from baseline in the CAPS‐5 severity score. Secondary outcomes were response rate, remission rate, participants with ≥1 treatment‐emergent adverse event (TEAE), participants with ≥1 severe TEAE, and suicidal ideation.

### 2.5 Risk of bias assessment

We assessed the risk of bias in the studies included using the revised Cochrane “Risk of bias” tool for randomized trials (RoB 2.0). Rob 2.0 deals with five specific domains: (1) bias arising from the randomization process; (2) bias due to deviations from intended interventions; (3) bias due to missing outcome data; (4) bias in the measurement of the outcome; and (5) bias in the selection of the reported result. Two review authors individually applied the tool to each included study. Disagreements were resolved by discussion to reach a common ground between the two review authors. If the matter remains unresolved, a third review author will act as a judge to give a final judgment.

### 2.6 Data synthesis and effect measures

Meta‐analysis was carried out using Review Manager (RevMan, version 5.4). A random‐effects model with the Der Simonian‐Laird variance estimator was used. We pooled our dichotomous outcomes as relative risk ratios (RR) and pooled our continuous outcomes as standard mean difference (SMD) with a 95% confidence interval for both types of outcomes. Heterogeneity was assessed using the *I* ^2^ index and chi‐square test. A *p*‐value of ≤0.1 was considered statistically significant. Interpretation of the *I* ^2^ values was done according to the Cochrane Handbook for Systematic Reviews of Interventions. Publication bias was checked using a funnel plot and Egger's regression test was used to assess the asymmetry of the funnel plot if the number of studies in a meta‐analysis was more than 10.

## 3 RESULTS

### 3.1 Study selection

**FIGURE 1:**
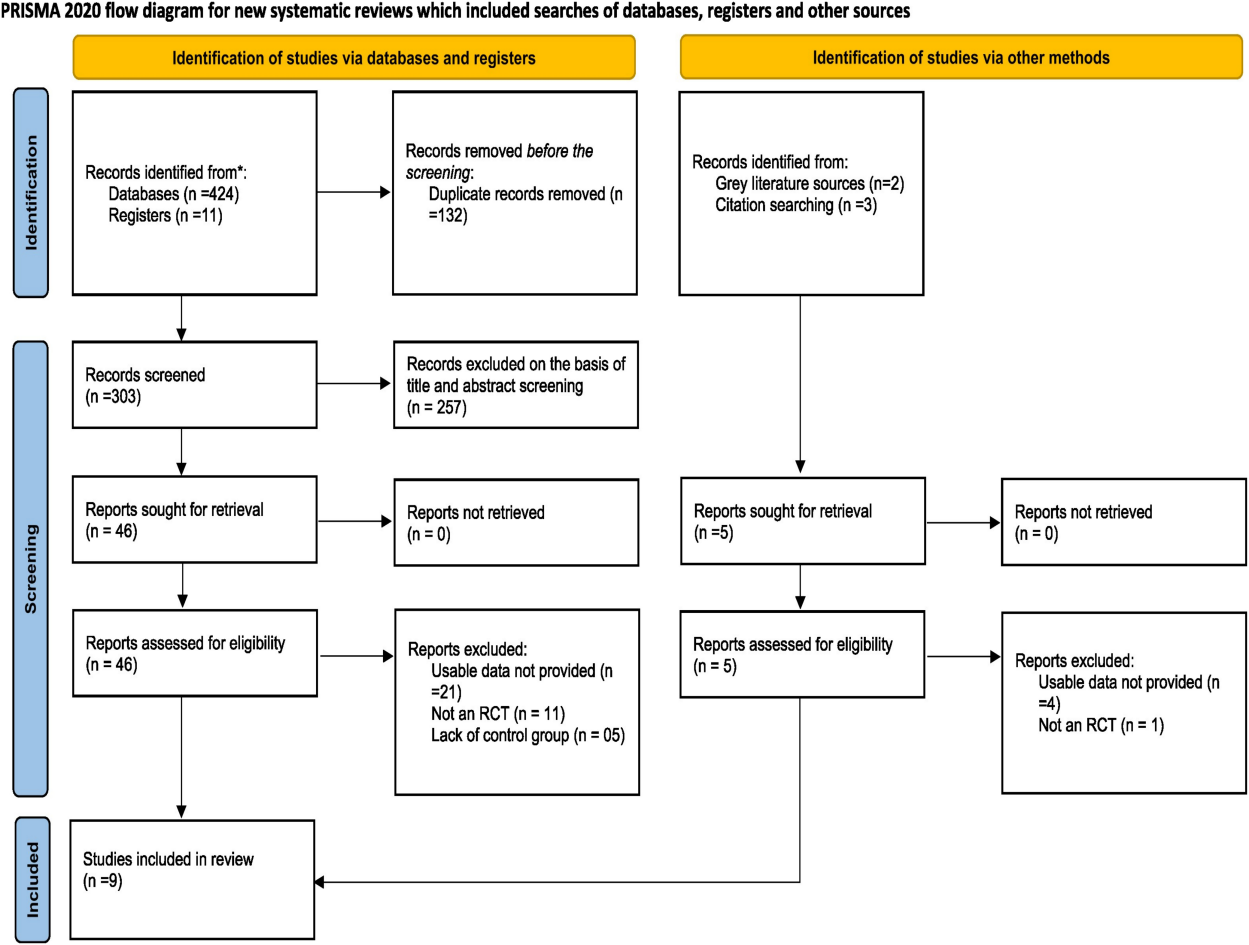
PRISMA 2020 flow chart of included and excluded trials. PRISMA, Preferred Reporting Items for Systematic Reviews and Meta‐Analyses.

A total of 424 studies were obtained from our preliminary database search. After screening, nine RCTs were found eligible according to our inclusion criteria. The study selection process is illustrated in the PRISMA Flowchart (Figure 1).

### 3.2 Study characteristics

Nine studies with a total of 297 participants with PTSD were included in this meta‐analysis.^22^, ^24^, ^30^, ^33^, ^34^, ^35^, ^36^, ^37^ Most of the studies (67%) had a follow‐up of 12 months. MDMA dosage in the intervention group varied from 50 mg to 125 mg across the included RCTs. The control group consisted of inactive doses of MDMA (25–40 mg) or placebo. The severity of PTSD was measured using CAPS‐4 and CAPS‐5 in eight out of nine studies while Bouso et al. used the Severity of Symptoms Scale for PTSD (SSSPTSD) for the diagnosis of PTSD.^33^ Most studies employed three sessions of MDMA‐AT. The detailed study characteristics are presented in Table 1.

**TABLE 1:** Study characteristics of included studies.

Study ID	Location	Trial design	Long term follow‐up	Number of participants	Age (years)	Male (%)	Intervention	Control	PTSD scale	Number of MDMA sessions
Mitchell 2023	USA and Israel	Multi‐site, double‐blind study	N/A	53 vs. 51	38.2 (11) vs. 40 (9.6)	39.6% vs. 17.6%	120 mg MDMA	Placebo	CAPS‐V	3 sessions
MP4	Canada	Single‐site, double‐blind study	12 months	4 vs. 2	47.7 (8.82)	3 (50%)	MDMA	Placebo	CAPS‐IV	2 sessions
Mithoefer 2018	USA	Triple‐blind study	12 months	12 vs. 7 vs. 7	40.7 (11.1) vs. 29.1 (4.0) vs. 39.2 (9.7)	8 (67) vs. 6 (86%) vs. 5 (71%)	125 mg MDMA	30 mg MDMA	CAPS‐IV	3 sessions
Mitchell 2021	USA, Canada, Israel	Randomized, double‐blind study	N/A	90 (46 vs. 44)	43.5 vs. 38.2	19% vs. 12%	80 mg MDMA	Placebo	CAPS‐V	3 sessions
Oehen 2013	Switzerland	Double‐blind study	12 months	8 vs. 4	42.1 (12.8) vs. 40.0 (6.2)	12% vs. 25%	125 mg MDMA	25 mg MDMA	CAPS‐IV	3 sessions
Mithoefer 2011	USA	Double‐blind study	2 months	12 vs. 8	40.2 (7.6) vs. 40.8 (7.0)	17% vs. 13%	125 mg MDMA	Placebo	CAPS‐IV	2 sessions
MP9	Israel	Triple‐blind study	12 months	10 (3 vs. 5)	3 (100%) vs. 5 (100%)	100%vs40%	125 mg MDMA	25 mg MDMA	CAPS‐IV	2 sessions
Bouso 2008	Spain	Double‐blind study	12 months	6	29 to 49	0% vs. 100% (female)	75 mg MDMA	Placebo	CAPS‐IV	N/A
Ot'alora 2018	USA	Double‐blind study	12 months	28 (22 vs. 6)	42.55 (13.3) vs. 40.0 (11.7)	36.6% vs. 16.7%	125 mg MDMA	40 mg MDMA	CAPS‐IV	2 sessions

### 3.3 Assessment of risk of bias

**FIGURE 2:**
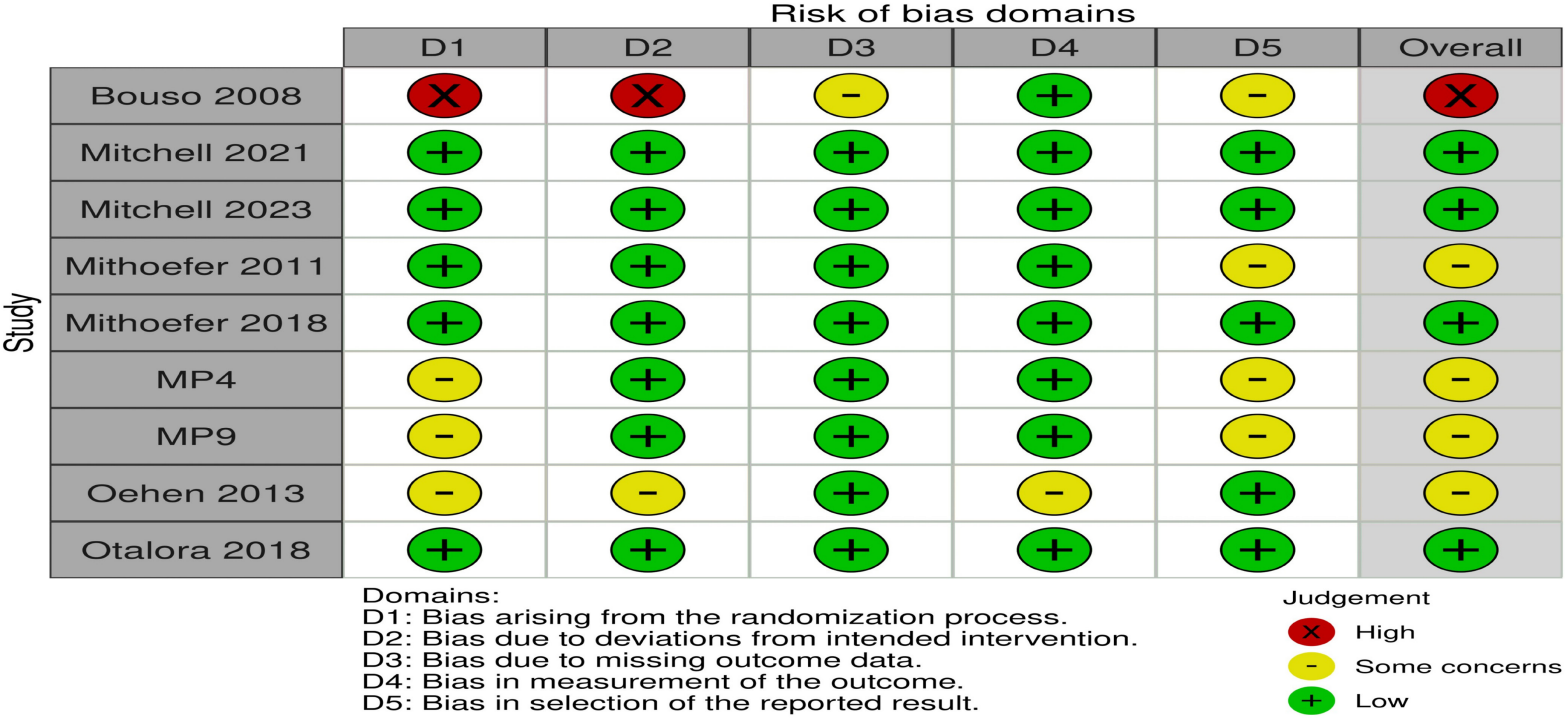
Quality assessment of randomized controlled trials (RCTs).

Only four studies were judged to be at a low risk of bias.^22^, ^24^, ^30^, ^35^ Four studies have some concerns of bias due to the lack of information regarding the pre‐specified analysis plan and problems arising during the randomization process.^34^, ^36^, ^37^ One study was found to be at a high risk of bias due to a lack of data about allocation concealment and pre‐specified analysis plan, and missing outcome data as the study was prematurely terminated (Figure 2).

### 3.4 Primary outcomes

#### 3.4.1 Change from baseline in CAPS‐5 severity score

**FIGURE 3:**
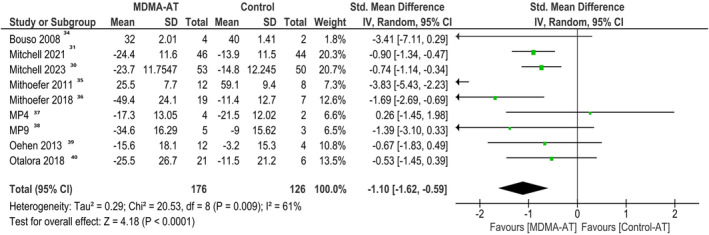
Forest plot of change from baseline in CAPS‐5 severity score.

Our meta‐analysis showed that MDMA‐AT led to a significant reduction in the CAPS‐5 severity scores as compared to the control group (SMD −1.10, 95% CI: −1.62 to −0.59; Figure 3) The estimated heterogeneity among the studies was moderate (*I* ^2^ = 61%).

#### 3.4.2 Secondary outcomes

##### Response rate

Our meta‐analysis demonstrated that more patients in the MDMA‐AT group exhibited significant responses as compared to patients in the control group (RR 1.59, 95% CI: 1.22, 2.08; Figure S1). Heterogeneity among the studies was found to be minimal (*I* ^2^ = 17%).

##### Remission rate

The remission rate was found to be significantly greater in the intervention group as compared to the control group (RR 2.32, 95% CI: 1.47 to 3.66; Figure S2) with minimal heterogeneity (*I* ^2^ = 0%).

##### ≥1 treatment‐emergent adverse events (TEAE)

This outcome was reported by only five studies. Our meta‐analysis found no significant difference regarding the incidence of ≥1 TEAE between the two groups (RR 1.03, 95% CI: 0.93 to 1.15; Figure S3). Statistical heterogeneity was found to be minimal (*I* ^2^ = 4%).

##### ≥1 severe TEAE

Our meta‐analysis of 6 RCTs found no significant difference regarding the incidence of ≥1 severe TEAE between the two groups (RR 0.95, 95% CI: 0.09 to 10.37; Figure S4). Heterogeneity among the studies was moderate (*I* ^2^ = 53%).

#### 3.4.3 Suicidal ideation

Suicidal ideation was reported by only four studies. We found no significant difference between the two groups regarding suicidal ideation (RR 1.17, 95% CI: 0.44 to 3.12; Figure S5). The inter‐study heterogeneity was found to be minimal (*I* ^2^ = 0%).

### 3.5 Subgroup analysis

When subgroup analysis was performed for the primary outcome based on the type of control group, no significant difference was found between the two groups (*p* = 0.65; Figure S6).

## 4 DISCUSSION

This study assessed the safety and efficacy of MDMA‐AT in treating patients with chronic refractory PTSD. The findings demonstrate that individuals who received MDMA in conjunction with psychotherapy exhibited higher remission and response rates compared to those who received placebo or inactive doses of MDMA. MDMA‐AT also led to a reduction in PTSD symptom severity, as evidenced by a decrease in the severity score on CAPS‐5 from baseline. The results did not show any significant difference between the two groups in terms of experiencing TEAE or severe TEAE, as well as suicidal ideation. These findings highlight the overall efficacy, tolerability, and safety of MDMA use in conjunction with psychotherapy in the treatment of PTSD.

Our findings on the superior efficacy of MDMA‐AT in treating PTSD compared to a placebo, align with previous systematic reviews.^25^, ^26^, ^27^, ^28^, ^29^ For instance, Smith et al. pooled outcomes from five RCTs and demonstrated that MDMA‐AT was more likely to yield a clinically significant reduction in PTSD symptoms and higher rates of remission compared to a placebo.^26^ Even when various comparators were employed, MDMA‐AT consistently exhibited greater effectiveness.^25^, ^27^, ^28^, ^29^ Our meta‐analysis provides more robust evidence concerning the efficacy of MDMA‐AT in the treatment of PTSD due to the inclusion of two relatively large RCTs weighing 48.1% and 36.5% respectively.^22^, ^30^ In both trials, a significant decrease in PTSD symptoms in the MDMA‐AT group as compared to the placebo group was noted. In one of these trials, 67% of participants in the MDMA‐AT group no longer met the diagnostic criteria for PTSD, compared to 33% in the placebo group. Additionally, 33% of participants in the MDMA‐AT group achieved remission, compared to only 5% in the placebo group.^22^

Several mechanisms have been postulated to elucidate the beneficial impact of MDMA in the treatment of PTSD. Existing research suggests that MDMA crosses the blood–brain barrier and binds to monoamine transporters for norepinephrine, dopamine, and serotonin.^18^, ^38^ This binding activity facilitates the reuptake of these neurotransmitters from the synaptic cleft and transports them across neuronal membranes, where they are stored in vesicles for subsequent utilization.^39^ More importantly, this binding induces the non‐vesicular transporter‐mediated release of serotonin, dopamine, and norepinephrine into the synaptic cleft.^40^, ^41^ This mechanism is believed to underlie the prosocial effects of MDMA in psychotherapy, manifesting as increased empathy, social closeness, inclusivity, and trust between patients and therapists.^18^, ^19^, ^20^ The elevation of serotonin resulting from MDMA ingestion is also found to promote oxytocin release, a hormone associated with enhanced empathy and other prosocial behaviors.^42^

Another pivotal mechanism by which MDMA complements psychotherapy in the context of PTSD involves its role in the extinction and consolidation of traumatic memories.^18^, ^19^, ^20^ MDMA accomplishes this by upregulating or restoring normal levels of brain‐derived neurotrophic factor (BDNF) in key brain regions such as the amygdala, ventromedial prefrontal cortex (vmPFC), and hippocampus. Adequate BDNF availability in these regions is crucial for memory consolidation and disrupting the association of fear‐inducing memories, thereby reducing emotional reactivity associated with traumatic experiences.^18^ This mechanism, coupled with the improved prosocial effects facilitating a stronger therapeutic alliance during therapy, is believed to contribute to the efficacy of MDMA in PTSD treatment.^18^

The results of the current meta‐analysis indicate that there is no significant difference in the incidence of adverse events and suicidal tendencies between individuals subjected to MDMA‐AT and those who were not, thereby signifying MDMA‐AT as generally safe and well‐tolerated. Although prior meta‐analyses did not undertake statistical evaluations of this facet, but relied on individual study accounts, they similarly concluded with MDMA‐AT safety profile in the treatment of PTSD.^25^, ^26^, ^27^ In one meta‐analysis, serious adverse events were documented in only one experimental study,^25^ while another noted a dose‐dependent escalation in adverse events,^26^ with depressive symptoms and suicidality being the most common adverse outcomes.^25^, ^26^ Conversely, a recent meta‐analysis reported a higher prevalence of adverse outcomes such as diminished mood, bruxism, and nausea in the experimental cohort compared to the placebo group.^29^ Caution, though, must be exercised in interpreting the later study due to encountered limitations such as the limited number of included studies and their respective small sample size, compromised blinding procedures, heterogeneity in depression outcome reporting, and the utilization of substantial MDMA doses.^29^

The literature also reported the superiority of MDMA‐AT in the treatment of PTSD compared to other approaches, such as psychopharmacology. Compared to antidepressants, specifically, sertraline and paroxetine, MDMA‐AT yielded a more significant decrease in CAPS scores; double that of paroxetine and triple that of sertraline. It also resulted in lower dropout rates and improved patient compliance.^17^ Better compliance with MDMA, aside from its therapeutic profile, may be attributed to its administration strategy. As compared to the chronic daily dosing of sertraline and paroxetine, MDMA is administered in a single dose in a controlled setting, spaced a month apart.^17^

In general, the accumulated evidence is congruent with the efficacy, tolerability, and overall safety of MDMA use as an adjunct to psychotherapy in PTSD treatment. Nonetheless, there are still some concerns regarding MDMA potential abuse and the literature is inconsistent in this regard. According to Pantoni's report, a dose of 3 mg/kg of MDMA might be the threshold amount, and anything greater than that might have the potential for addictive effects.^38^ Mitchell et al. demonstrated that MDMA didn't have any abuse potential at a dose of 120 mg after the first experimental session.^22^ Other researchers demonstrated that MDMA imposes minimal risk of substance abuse and neurocognitive decline^43^ or that MDMA does not share the same risk profile as recreational ecstasy.^44^ These contrasting opinions warrant additional RCTs to establish MDMA's potential addictive effects.

### 4.1 Strengths and limitations

There are several strengths inherent in this study. First, the statistical robustness of the conducted analysis exceeds that of previous meta‐analyses, owing to the inclusion of a larger number of RCTs and correspondingly larger sample sizes. More specifically, two recent RCTs by Mitchell et al., with 104 and 90 participants, respectively,^22^, ^30^ accounted for 65% of the total participants in our study. In contrast, previous meta‐analyses reported sample sizes ranging from 85^29^ to 18^26^ participants. Secondly, meticulous study screening and selection were performed utilizing multiple reputable databases to maximize the inclusion of all relevant studies.

Several limitations warrant consideration in the interpretation of current findings. This review relied on aggregate‐level data analysis, prompting inquiry into whether results would differ from those addressing patient‐level data. This study also lacked subgroup analysis due to the unavailability of data and no outcomes were assessed after follow‐up. The follow‐up assessment could not be conducted due to the crossover of participants from the control group to the intervention group during the trial's implementation. In our study, outcome values were estimated before this point, which presents a limitation due to the inability to evaluate long‐term outcomes. However, this approach enhances the validity of our findings, as they only reflect the true outcomes of the intervention group.

Another shortcoming of the current meta‐analysis stems from the considerable heterogeneity observed among the included studies. Pertaining to the assessment of PTSD symptom severity, some studies utilized the CAPS‐5, while others relied on the CAPS‐4. Participant demographics encompassed diverse backgrounds in terms of geographical distribution, gender and age representation, as well as associated comorbidities. For instance, comorbid anxiety and depression were documented in four and six RCTs, respectively. These studies did not specify whether participants were undergoing treatment for these comorbid conditions at the time of the trial. Several confounders were also noted which may question the validity of MDMA‐AT efficacy in PTSD treatment, including variations in prior MDMA usage and previous PTSDS treatment. In the included RCTs, MDMA usage ranged from never^37^ to lifetime use,^22^, ^30^ and previous PTSD treatment was reported in five out of nine RCTs. Heterogeneity was also evident in the MDMA‐AT intervention, including the number of treatment sessions administered, MDMA dosage, and short‐ and long‐term follow‐up durations. While some RCTs conducted two treatment sessions, others administered a total of three. The dosage of MDMA in the included trials ranged from as low as 50 mg to 125 mg. Furthermore, some RCTs such as Oehen et al.^37^ failed to report adverse events associated with MDMA‐AT intervention and placebo administration. A final limitation concerns the blinding of participants to their group assignment. Despite the inclusion of only RCTs, both physicians and patients in the majority of the trials were likely aware of the treatment assignment due to the noticeable effects produced by MDMA‐AT. Future investigations are warranted to rigorously estimate the effectiveness and safety of MDMA in the treatment of PTSD taking into consideration the aforementioned limitations.

## 5 CONCLUSION

Our analysis has demonstrated that when administered in a controlled and professional setting, MDMA, through its therapeutic and safety profile, translates effectively to treating chronic, treatment‐resistant PTSD. The findings of this study have significant implications for the treatment of PTSD and suggest that MDMA‐AT may be a promising therapeutic approach for individuals who have not responded to traditional PTSD treatments. Moreover, this research underscores the established tolerability and reasonably safe profile of MDMA‐AT. Nonetheless, considering the aforementioned limitations inherent in this meta‐analysis, there remains a necessity for future investigations to enhance confidence in the efficacy and safety of MDMA‐AT in PTSD treatment. Subsequent research endeavors should explore whether the efficacy and occurrence of adverse events are influenced by MDMA dosage and participant characteristics, as well as the severity of PTSD symptoms. Standardization of PTSD assessment methods is imperative to facilitate inter‐study comparisons and yield more robust findings. Furthermore, given that the majority of clinical trials have primarily focused on short‐term MDMA‐AT therapy, there is a clear need for additional longitudinal studies to elucidate the long‐term effectiveness of MDMA‐AT treatment.

## AUTHORS CONTRIBUTIONS

ME conceived the idea and ME along with GS established a search strategy. KS, SE, and MHK retrieved the articles and screened them for relevance. JJ, KS, and HM assisted with full text screening. GS, SIH, and DS then ran quality assessment on the selected articles. Data was extracted by SE, MHK, JJ, KS, and HM. GS, KS, ME, and AJN proofread the extracted data. SIH, DS, KS, and GS then ran the meta‐analysis. All the authors contributed to the initial and final draft of the article. All authors read and approved the final version of this article.

## FUNDING INFORMATION

No financial support was received for this study.

## CONFLICT OF INTEREST STATEMENT

The authors declare no conflict of interest.

## ETHICS STATEMENT

Approval of the Research Protocol by an Institutional Reviewer Board: N/A.

Informed Consent: N/A.

Registry and Registration No. of the Study/Trial: N/A.

Animal Studies: N/A.

We confirm that we have read the Journal's position on issues involved in ethical publication and affirm that this report is consistent with those guidelines.

## PROSPERO REGISTRATION NUMBER

CRD42023394142.

## Supporting information


Appendix S1

## Data Availability

We have provided the raw data in the Appendix S1.
