# Supplementary material for: MDMA‐assisted psychotherapy for the treatment of PTSD: A systematic review and meta‐analysis of randomized controlled trials (RCTs)
Source: Neuropsychopharmacol Rep. 2024 Oct 9;44(4):672–81. doi: 10.1002/npr2.12485 (PMC11609750; doi:10.1002/npr2.12485)
Supplement: Supplementary file 1 — Appendix S1 [file NPR2-44-672-s001.docx]

# Supplementary Material

**MDMA-assisted psychotherapy for the treatment of PTSD: A Systematic Review and Meta-analysis of Randomized Controlled Trials (RCTs)**

This supplemental material has been provided by the authors to give readers additional information about their work.

**Supplementary Table 1: Search Strategy for MEDLINE**

| **Number** | **Search Terms** |
| --- | --- |
| #1 | MDMA [All Fields] |
| #2 | p N-Methyl-3,4-methylenedioxyamphetamine [All Fields] |
| #3 | “Ecstasy” [All Fields] |
| #4 | 3,4-Methylenedioxyamphetamine [All Fields] |
| #5 | MDMA-assisted psychotherapy [All Fields] |
| #6 | MDMA-AT [All Fields] |
| #7 | #1 OR #2 OR #3 OR #4 OR #5 OR #6 |
| #8 | PTSD [All Fields] |
| #9 | Posttraumatic stress disorder [All Fields] |
| #10 | #8 OR #9 |
| #11 | #7 AND #10 |

**Supplementary Figure 1. Forest plot of response rate**

**
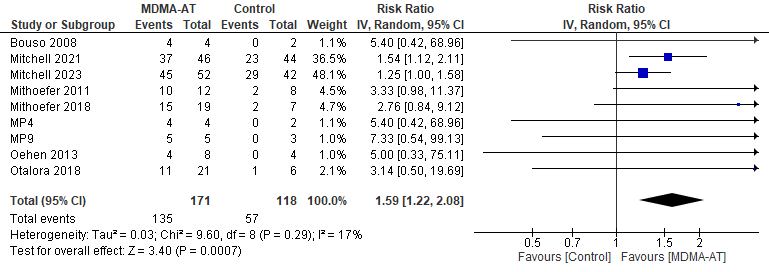
**

**Supplementary Figure 2. Forest plot of remission rate**

**
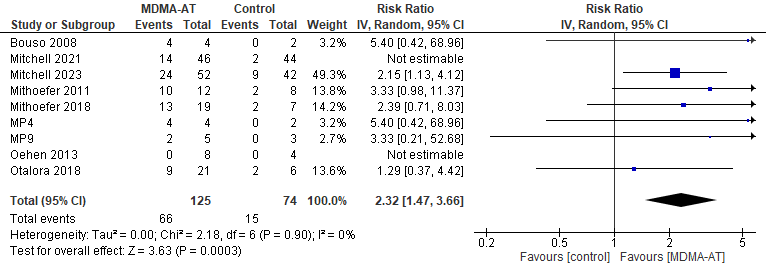
**

**Supplementary Figure 3. Forest plot of ≥1 TEAE**

**
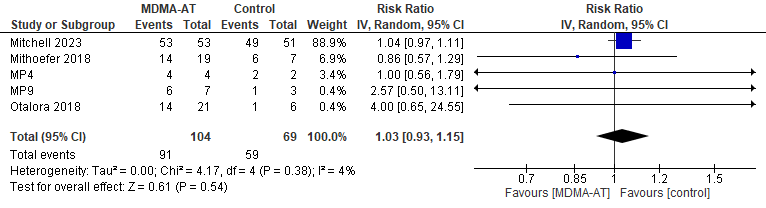
**

**Supplementary Figure 4. Forest plot of ≥1 severe TEAE**

**
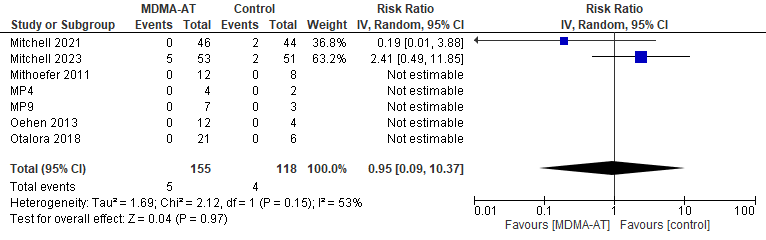
**

**Supplementary Figure 5. Forest plot of suicidal ideation**

**
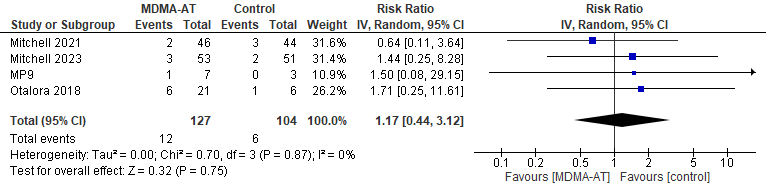
**

**Supplementary Figure 6. Forest plot of subgroup analysis of change from baseline in CAPS-5 severity score**

**
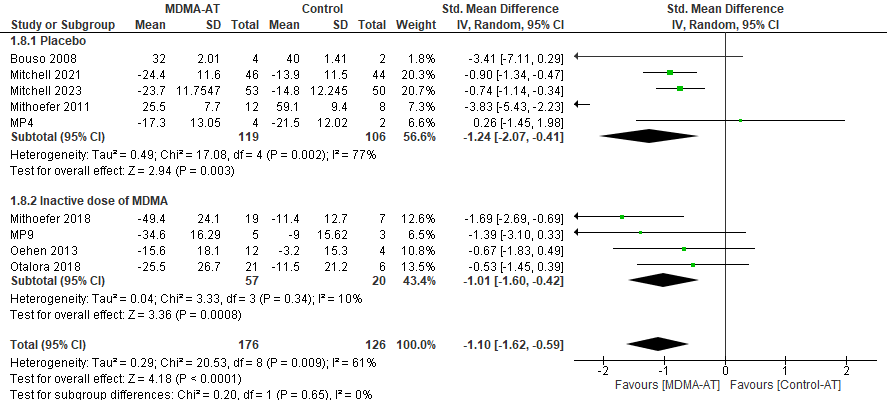
**

**Raw data (Outcome extraction)**

| **Study ID** | **Trial name** | **Responders** | | | | **Remission** | | | | **Participants with ≥1 TEAE** | | | | **Participants with ≥1 severe TEAE** | | | | **Suicidal ideation** | | | | **Change from baseline in CAPS-5 Severity score** | | | | | |
| --- | --- | --- | --- | --- | --- | --- | --- | --- | --- | --- | --- | --- | --- | --- | --- | --- | --- | --- | --- | --- | --- | --- | --- | --- | --- | --- | --- |
|  |  | **MDMA** | | **Control** | | **MDMA** | | **Control** | | **MDMA** | | **Control** | | **MDMA** | | **Control** | | **MDMA** | | **Control** | | **MDMA** | | | **Control** | | |
|  |  | **Events** | **Total** | **Events** | **Total** | **Events** | **Total** | **Events** | **Total** | **Events** | **Total** | **Events** | **Total** | **Events** | **Total** | **Events** | **Total** | **Events** | **Total** | **Events** | **Total** | **Mean** | **SD** | **Total** | **Mean** | **SD** | **Total** |
| **Bouso 2008** | - | 4 | 4 | 0 | 2 | 4 | 4 | 0 | 2 | - | - | - | - | - | - | - | - | - | - | - | - | 32.0 | 2.01 | 4 | 40.0 | 1.41 | 2 |
| **Mitchell et al 2023** | NCT04077437 | 45 | 52 | 29 | 42 | 24 | 52 | 9 | 42 | 53 | 53 | 49 | 51 | 5 | 53 | 2 | 51 | 3 | 53 | 2 | 51 | -23.7 | 11.7547 | 53 | -14.8 | 12.245 | 50 |
| **Mitchell 2021** | NCT03537014 | 37 | 46 | 23 | 44 | 14 | 46 | 2 | 44 | - | - | - | - | 0 | 46 | 2 | 41 | 2 | 46 | 3 | 44 | -24.4 | 11.6 | 46 | -13.9 | 11.5 | 44 |
| **Mithoefer 2011** | NCT00090064 | 10 | 12 | 2 | 8 | 10 | 12 | 2 | 8 | - | - | - | - | 0 | 12 | 0 | 8 | - | - | - | - | 25.5 | 7.7 | 12 | 59.1 | 9.4 | 8 |
| **mithoefer.et.al 2018** | NCT01211405 | 15 | 19 | 2 | 7 | 13 | 19 | 2 | 7 | 14 | 19 | 6 | 7 | - | - | - | - | - | - | - | - | -49.4 | 24.1 | 19 | -11.4 | 12.7 | 7 |
| **MP4** | NCT01689740 | 4 | 4 | 0 | 2 | 4 | 4 | 0 | 2 | 4 | 4 | 2 | 2 | 0 | 4 | 0 | 2 | - | - | - | - | -17.3 | 13.05 | 4 | -21.5 | 12.02 | 2 |
| **MP9** | NCT01958593 | 5 | 5 | 0 | 3 | 2 | 5 | 0 | 3 | 6 | 7 | 1 | 3 | 0 | 7 | 0 | 3 | 1 | 7 | 0 | 3 | -34.6 | 16.29 | 5 | -9.0 | 15.62 | 3 |
| **Oehen 2013** | NCT00353938 | 4 | 8 | 0 | 4 | 0 | 8 | 0 | 4 | - | - | - | - | 0 | 12 | 0 | 4 | - | - | - | - | -15.6 | 18.1 | 12 | -3.2 | 15.3 | 4 |
| **Otalora 2018** | NCT01793610 | 11 | 21 | 1 | 6 | 9 | 21 | 2 | 6 | 14 | 21 | 1 | 6 | 0 | 21 | 0 | 6 | 6 | 21 | 1 | 6 | -25.5 | 26.7 | 21 | -11.5 | 21.2 | 6 |
